# The mRNA-based RSV vaccine mRNA-1345 induces robust and durable CD4^+^ T cell responses against RSV in younger and older adults

**DOI:** 10.1126/sciadv.aeh4436

**Published:** 2026-10-21

**Authors:** Anthony DiPiazza, Emily Marcisak, Wen-Han Yu, Daniel Makrinos, Hsiao-Hsuan Kuo, Jenna Landy, Shannon McGrath, Xiaolin Chang, Maria Cavallaro, Christopher Wu, Adam Essene, Haining Lin, Laura Walker, Andrea Carfi, Jaap Oostendorp, Robert Paris, Yanbo Sun

**Affiliations:** Moderna Inc., Cambridge, MA, USA.

## Abstract

Respiratory syncytial virus (RSV) remains a health threat, particularly for infants, older adults, and immunocompromised individuals. Although neutralizing antibodies can help limit infection and viral replication, T cells are essential for reducing disease severity. Assessment of age-related declines in T cell function are important for vaccine evaluation. We used intracellular cytokine staining, activation-induced marker, and Meso Scale Discovery assays and single-cell multiomic profiling to evaluate RSV vaccine mRNA-1345 responses in younger and older adults. mRNA-1345 induced comparable CD4^+^ and CD8^+^ T cell responses in both cohorts, with CD4^+^ responses persisting for 24 months. The CD4^+^ response was strongly T helper 1 (T_H_1)–polarized, with increased interferon-γ, interleukin-2, and tumor necrosis factor–α and minimal T_H_2 cytokines. Both CD4^+^ and CD8^+^ T cells showed increased polyfunctionality that remained elevated at 2 years. Single-cell and T cell receptor repertoire analyses showed similar Pre-F–specific phenotypes, transcriptional profiles, and clonal overlap across age groups, supporting robust, durable cellular immunity after mRNA-1345 vaccination. These findings indicate shared vaccine-elicited T cell immunity in adults.

## INTRODUCTION

Respiratory syncytial virus (RSV) remains a public health concern, particularly among infants, older adults, and immunocompromised individuals globally (*1*–*4*). RSV is a leading cause of lower respiratory tract infections, contributing to 3.6 million hospitalizations and over 100,000 deaths annually, predominantly in children under 5 years of age and in older individuals (*5*–*8*). Although three RSV vaccines have recently been approved for medical use in the United States (*9*), challenges remain in understanding how the immune response in older adults compares to that in younger populations, both in terms of similarities and critical differences potentially associated with immunosenescence.

The immune response to RSV is complex (*10*–*19*). Although neutralizing antibodies play a critical role in protecting against initial infection and reducing viral load (*20*–*23*), T cells are increasingly recognized as essential for viral clearance and disease resolution (*24*–*26*). Evidence from both clinical and animal studies suggests that T cells, particularly CD8^+^ cytotoxic T cells and CD4^+^ T helper 1 (T_H_1) cells, contribute to the control of RSV infection and mitigation of disease severity (*24*, *27*–*32*). These T cells target RSV-infected cells, reducing viral replication and limiting the spread of the virus within the respiratory tract. Moreover, the presence of robust memory T cell response has been associated with reduced severity of subsequent RSV infections, highlighting their role in long-term protection (*33*).

T cell function is known to decline with age, a phenomenon referred to as immunosenescence (*34*). This age-related decline in immune function poses a major challenge in the context of RSV infection, as older adults are particularly vulnerable to severe RSV disease (*7*). Immunosenescence is characterized by reduced T cell proliferation, impaired cytokine production, and diminished cytotoxic activity (*34*–*37*). Furthermore, the aged immune system also exhibits a skewed T cell repertoire with decreased clonal diversity, leading to a less effective response to previously unidentified and recurring pathogens (*38*–*40*). Hence, it is crucial to understand how T cell responses differ across age groups to inform the design of RSV vaccines.

To comprehensively assess how the quality of the T cell response to vaccination may differ between older and younger adults, it is critical to investigate the transcriptional and clonal differences in T cell responses, in addition to the magnitude, across age groups. Transcriptional profiling can reveal age-related changes in gene expression that affect T cell function and longevity, such as alterations in signaling pathways, differentiation status, and metabolic fitness. Clonal analysis can provide insight into the diversity and dynamics of the T cell response, elucidating how aging shapes the T cell repertoire in response to vaccination. Together, these analyses provide a comprehensive network to understand how aging influences the quality and durability of vaccine-induced T cell immunity.

In this study, we evaluated T cell responses to mRNA-1345, an mRNA-based RSV vaccine that encodes a membrane-anchored RSV-F glycoprotein stabilized in the prefusion conformation (Pre-F), in older and younger adults to assess the impact of aging on the magnitude and polyfunctionality of vaccine-induced T cell immunity and the potential implications for protection against RSV. Furthermore, we examined transcriptional and clonal differences in T cell responses across age groups to understand how these factors may influence the quality and durability of the T cell response to mRNA-1345 vaccination and provide additional insights into age-related differences in vaccine-induced T cell immunity.

## RESULTS

### mRNA-1345 elicits comparably robust T cell responses in younger and older adults

To determine whether mRNA-based vaccines could induce comparable T cell response in younger and older adults, we first profiled the magnitude and kinetics of interferon-γ^+^ (IFN-γ^+^) CD4^+^ and CD8^+^ T cell responses in healthy younger (aged 18 to 49) and older (aged 50 to 75) adults following mRNA-1345 vaccination. Peripheral blood mononuclear cells (PBMCs) from days 1 (baseline), 8, 15, 29, 91, 181, 361, 541, and 721 after vaccination were analyzed using a T cell intracellular cytokine staining (ICS) assay (Fig. 1, fig. S1, and tables S1 and S2).

**Fig. 1.**
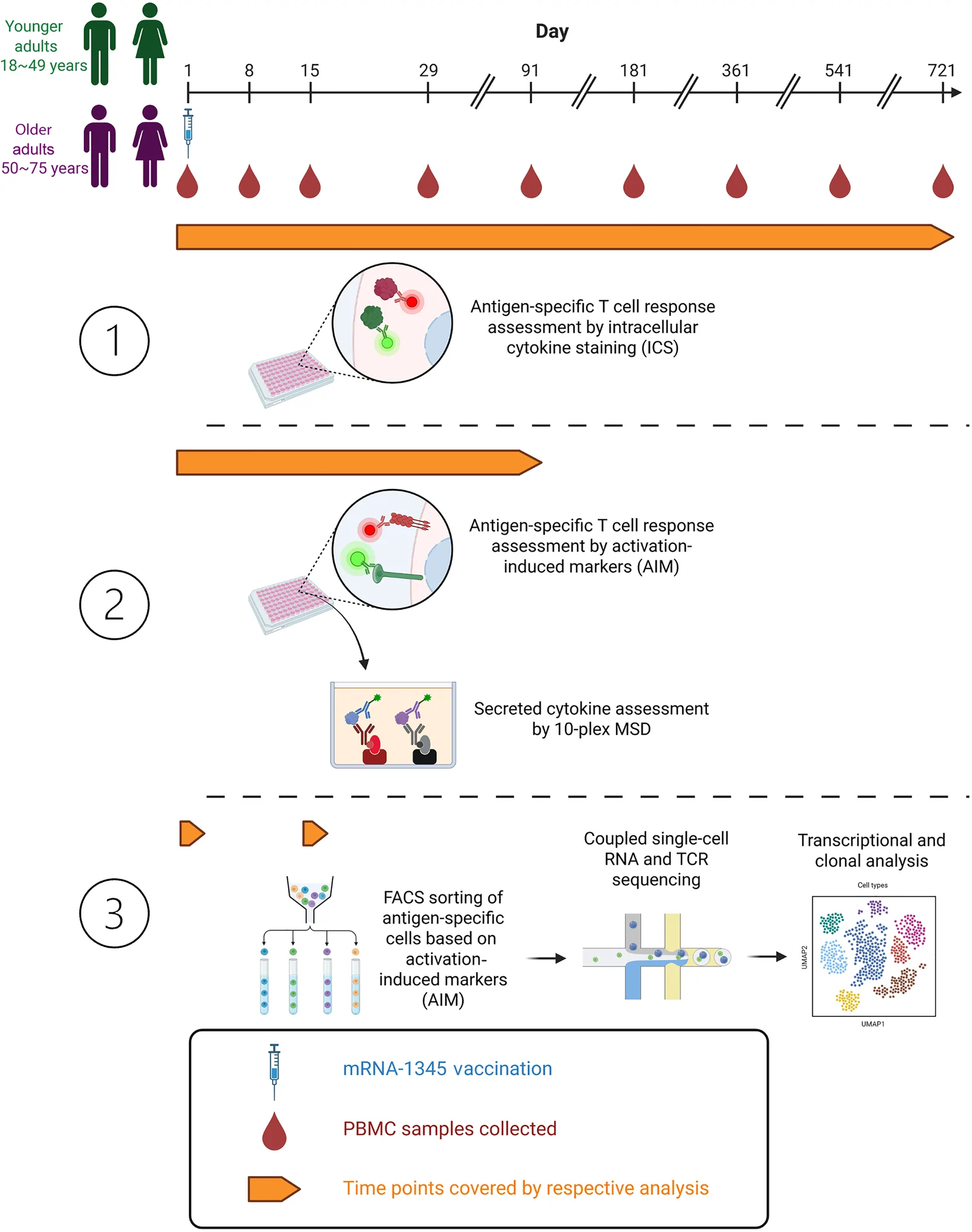
Study design. Created in BioRender. Walker, L. (2026) https://BioRender.com/a4bejg4.

Both IFN-γ^+^ CD4^+^ and CD8^+^ T cell response were robustly induced in both age groups following mRNA-1345 immunization (Fig. 2, A and B). Furthermore, the magnitudes of IFN-γ^+^ CD4^+^ and CD8^+^ T cell responses were comparable between younger and older individuals across all time points evaluated (fig. S2, A and B). To account for individual variability in baseline T cell response against Pre-F, we next assessed the percentage of responders by either IFN-γ^+^ CD4^+^ or IFN-γ^+^ CD8^+^ T cell response in each age group. Responders were defined as subjects who demonstrated IFN-γ responses ≥2-fold over baseline or those who had IFN-γ responses below the lower limit of detection (LLOD) at baseline but mounted IFN-γ responses ≥LLOD following vaccination. Using these criteria, we observed that mRNA-1345 elicited a high percentage of CD4^+^ T cell responders, with response rates exceeding 80% at 1 year after vaccination and remaining above 70% through day 721 in both age groups (Fig. 2A). Moreover, CD8^+^ T cell responder rates were also robust across age groups but waned over time (Fig. 2B). These findings underscore the ability of mRNA-1345 to induce strong cellular immune response with CD4^+^ T cell response showing satisfactory durability.

**Fig. 2.**
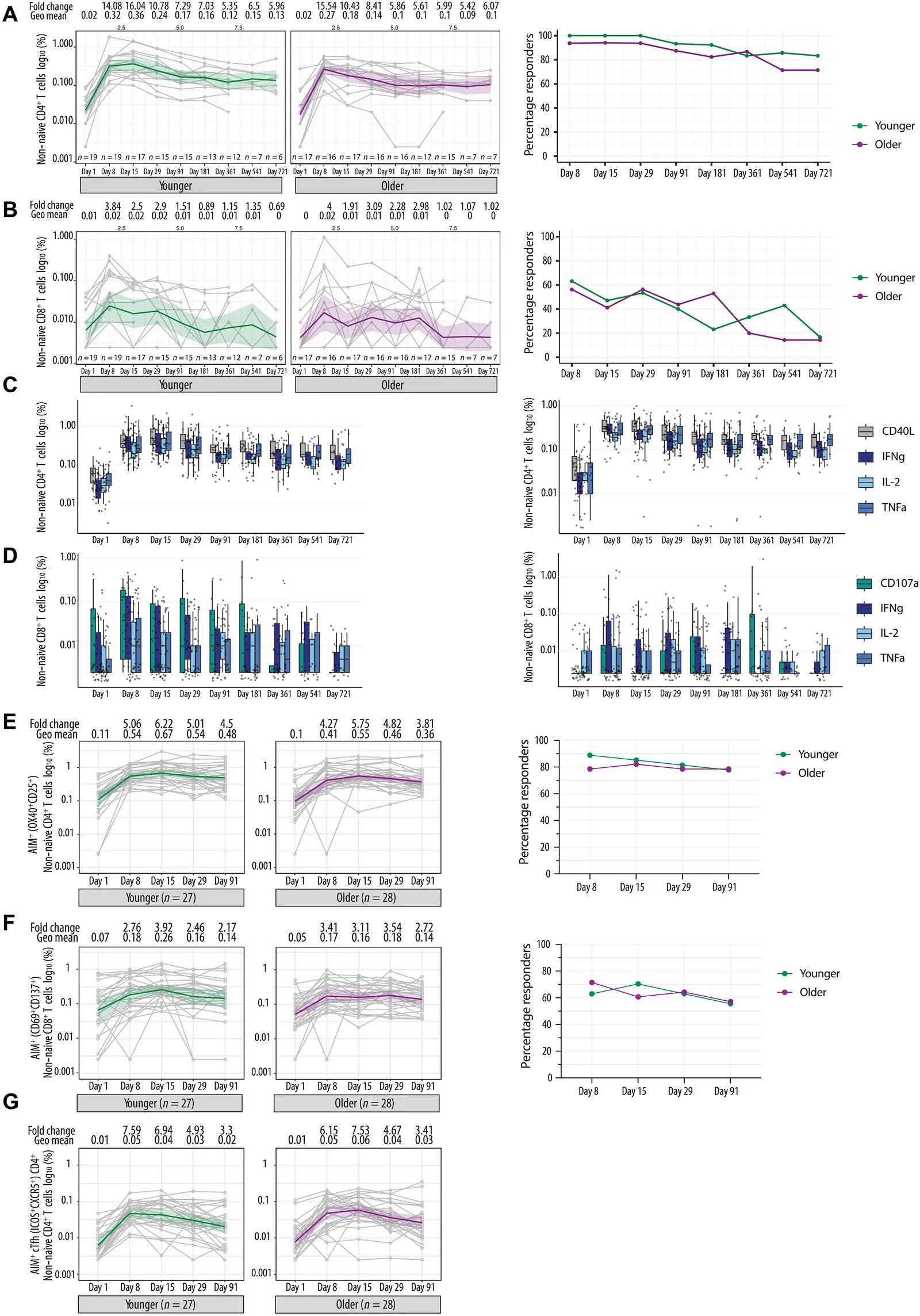
mRNA-1345 elicited robust and comparable CD8^+^ and CD4^+^ T cell responses in younger and older individuals throughout the study duration. (**A** and **B**) IFN-γ^+^ CD4^+^ (A) and CD8^+^ (B) T cell response by ICS. (Left) IFN-γ^+^ CD4^+^ and CD8^+^ T cells as a percentage of the parental non-naïve CD4^+^ or CD8^+^ T cell population. (Right) Percentage of responders (≥2-fold over baseline or ≥LLOD after vaccination if baseline was below the LLOD) at each time point. (**C** and **D**) All T_H_1 CD4^+^ (C) and CTL CD8^+^ (D) T cell cytokines evaluated by ICS. (Left) Younger adults. (Right) Older adults. (**E** and **F**) Pre-F–specific CD4^+^ (E) and CD8^+^ (F) T cell response by AIM. (Left) AIM^+^ cells defined as OX40^+^CD25^+^CD4^+^ and CD69^+^CD137^+^ CD8^+^, as a percentage of the non-naïve CD4^+^ or CD8^+^ T cell population. (Right) Percentage of responders at each time point. (**G**) Pre-F–specific cT_FH_ CD4^+^ (CXCR5^+^ ICOS^+^) T cell response by AIM (OX40^+^CD25^+^). In (A), (B), and (E) to (G), gray lines denote individual participants; bold lines and the shaded areas represent the geometric means and the 95% CIs for each group. In (C) and (D), dots represent an individual participant; boxes with bold lines represent the geometric means and the 95% CIs. The *y* axis was log_10_ transformed.

In addition to IFN-γ, we assessed other T_H_1 and cytotoxic lymphocyte (CTL) cytokines such as tumor necrosis factor–α (TNF-α) and interleukin-2 (IL-2), as well as activation markers CD40L and CD107a, and observed consistent kinetics and hierarchy at all time points studied (Fig. 2, C and D). Notably, frequencies of CD4^+^ T cells expressing any T_H_1 cytokines remained elevated above baseline in both younger and older individuals at 2 years postvaccination, indicating durable maintenance of polyfunctional T_H_1 response that is not impaired by aging (Fig. 2C).

Younger adults (aged 18 to 49) and older adults (aged 50 to 75) were enrolled at a 1:1 ratio and received a single dose of mRNA-1345 on day 1. PBMC samples were collected on days 1, 8, 15, 29, 91, 181, 361, 541, and 721. Workstream 1: Days 1, 8, 15, 29, 91, 181, 361, 541, and 721 PBMC samples were subjected to stimulation with the Pre-F peptide pool followed by ICS analysis to assess the magnitude of RSV Pre-F–specific T cell response. Workstream 2: Days 1, 8, 15, 29, and 91 PBMC samples were subjected to stimulation with the Pre-F peptide pool. The cells were used for activation-induced marker (AIM) analysis as an alternative method for detection of Pre-F–specific T cell response, whereas the culture supernatant was analyzed by 10-plex Meso Scale Discovery (MSD) assay for secreted cytokines. Workstream 3: Day 15 PBMC samples were subjected to fluorescence-activated cell sorting (FACS) based on AIM positivity followed by coupled single-cell RNA and T cell receptor (TCR) sequencing.

To provide a more comprehensive assessment of mRNA-1345–induced cellular immunity, we next evaluated the overall magnitude of Pre-F–specific as “any T_H_1” CD4^+^ T cell response and “any CTL” CD8^+^ T cell response. “Any T_H_1” CD4^+^ T cell responses were assessed by Boolean gating of IFN-γ, TNF-α, IL-2, and CD40L, capturing all Pre-F–specific T_H_1 CD4^+^ T cells expressing at least one of these cytokines or activation markers. Similarly, “any CTL” CD8^+^ T cell responses were assessed by Boolean gating of IFN-γ, TNF-α, IL-2, and CD107a, thereby encompassing the full spectrum of Pre-F–specific CTL CD8^+^ T cells with heterogeneous functional profiles. Using this comprehensive analysis, we observed that the magnitudes of both “any T_H_1” CD4^+^ T cell and “any CTL” CD8^+^ T cell responses were robust and comparable between younger and older adults at all time points analyzed (fig. S2, C and D). These findings further support that mRNA-1345 induces robust and polyfunctional T_H_1 CD4^+^ and CD8^+^ T cell immunity in both age cohorts.

To complement our ICS analysis and further validate the cellular immune response elicited by mRNA-1345 vaccination, we used a second flow cytometry–based assay—the AIM assay—on a subset of samples (Fig. 1 and tables S1 and S3). This method identifies antigen-specific T cells based on the up-regulation of surface markers associated with TCR engagement, providing an orthogonal measurement of antigen-specific T cell response. We used the AIM assay for three primary objectives (i) to provide a complementary assessment of magnitude and kinetics of CD4^+^ and CD8^+^ T cell response across age groups to our ICS analysis; (ii) to characterize circulating T follicular helper cells (cT_FH_ cells), an emerging correlate of antibody response to natural infections and vaccinations (*41*, *42*); and (iii) to enable FACS sorting of Pre-F–specific CD4^+^ and CD8^+^ T cells for downstream multiomic analysis. Using well-established AIM combinations (*43*–*48*), we observed that both Pre-F–specific CD4^+^ (OX40^+^CD25^+^) and CD8^+^ (CD69^+^CD137^+^) T cell responses were robust and comparable between younger and older adults and persisted until day 91, which is the latest time point evaluated by AIM (Fig. 2, E and F). Notably, CD4^+^ T cell responder rates remained high, with ∼80% responders at day 91 after vaccination across age groups (Fig. 2E). Similarly, CD8^+^ T cell responder rates were sustained at around 60% in both younger and older adults at day 91 (Fig. 2F). In addition, robust induction of cT_FH_ cells was detected in both age groups, with responses remaining elevated at day 91 and showing no notable difference between younger and older cohorts (Fig. 2G). These findings confirm the strong and sustained induction of key T cell subsets by mRNA-1345, as measured by orthogonal assay platforms.

Overall, our data suggest that mRNA-1345 elicits robust cellular immune responses, with the CD4^+^ T cell response demonstrating long-term durability across age groups, reinforcing its immunogenicity in both younger and older adult populations.

### mRNA-1345 induces a strong T_H_1-polarized CD4^+^ T cell response across age groups with minimal T_H_2 signature

Given the association between dysregulated balance of T_H_1/T_H_2 CD4^+^ T cell responses and severe RSV disease in clinical cases (*49*–*51*) and preclinical animal models (*51*–*53*), we assessed the polarization of mRNA-1345–induced CD4^+^ T cell response in both age cohorts. To determine the T_H_1 versus T_H_2 profile of CD4^+^ T cells induced by mRNA-1345, we used both ICS and MSD U-plex assays, a sandwich immunoassay for detection of soluble analytes (Fig. 1). These assays provide complimentary and orthogonal measurement of T_H_2-polarized CD4^+^ T cells at the single-cell level, as well as quantification of secreted T_H_2 cytokines in culture supernatants.

We observed minimal evidence of a T_H_2-biased response in both age groups and in both assays (Fig. 3, A and B). The frequency of IFN-γ^+^ CD4^+^ T cells was more than 30-fold higher than that of IL-4^+^, IL-5^+^, or IL-13^+^ CD4^+^ T cells at the peak of response (Figs. 2A and 3A). Consistently, T_H_1 cytokines were robustly produced, with IFN-γ concentrations exceeding T_H_2 cytokines by more than three orders of magnitude (Fig. 3, B and C), confirming the strong T_H_1 polarization of the CD4^+^ T cell response induced by mRNA-1345. Notably, IL-4–producing CD4^+^ T cells were detected by ICS at very low frequencies, but the levels of secreted IL-4 were below the LLOD across all donors by MSD (Fig. 3, A and B). In addition, we evaluated the levels of eotaxin, IL-10, and IL-17, which have also been associated with severe RSV symptoms (*54*–*57*) by MSD, and observed minimal production of these factors (Fig. 3D).

**Fig. 3.**
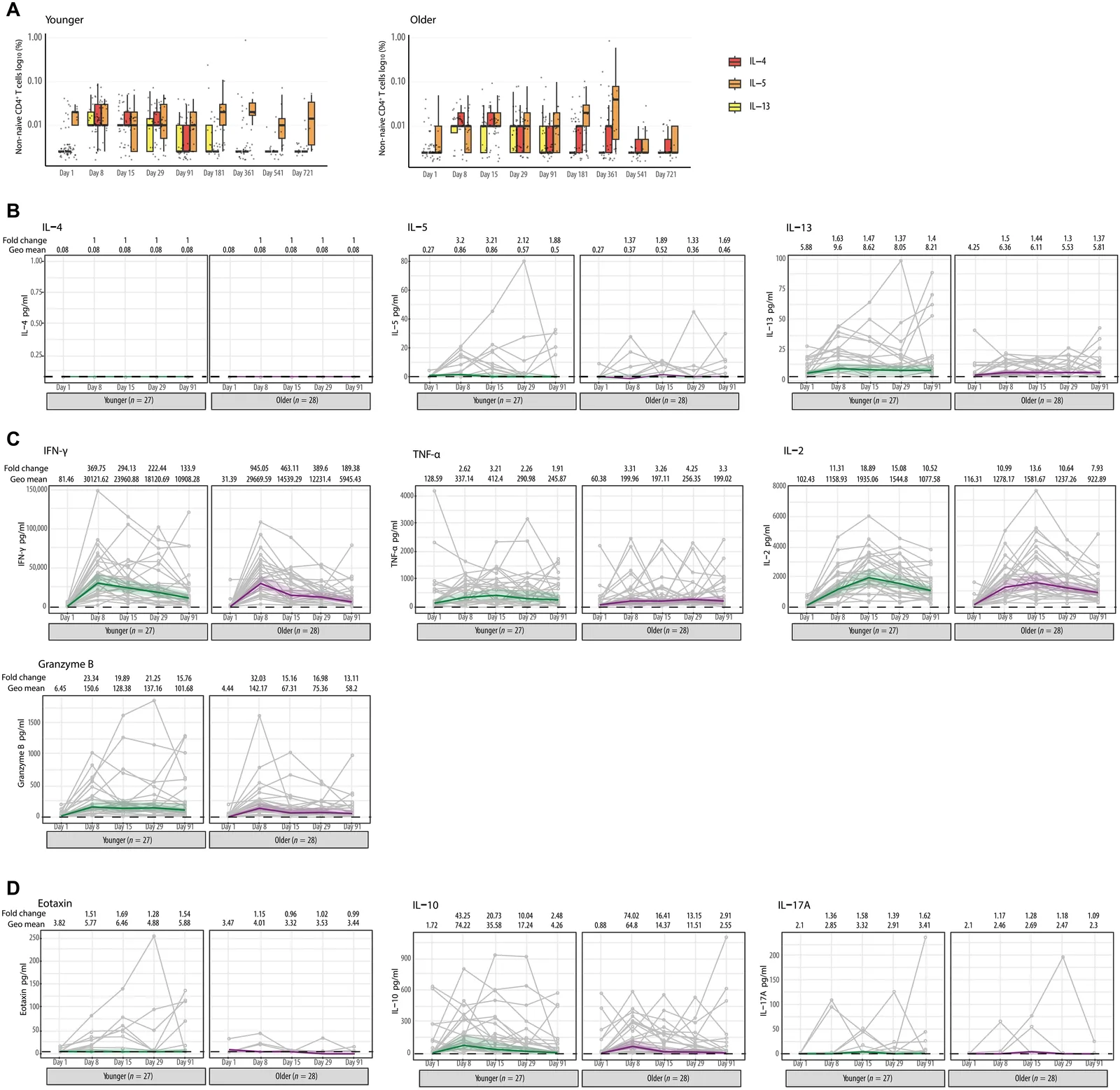
mRNA-1345 induced a strong T_H_1-polarized CD4^+^ T cell response with minimal T_H_2 involvement. T_H_1 versus T_H_2 T cell response against Pre-F following mRNA-1345 vaccination. PBMC samples were collected on days 1, 8, 15, 29, 91, 181, 361, 541, and 721; T_H_1 and T_H_2 CD4^+^ T cell responses were evaluated using ICS and MSD assays. (**A**) All T_H_2 CD4^+^ T cell cytokines evaluated by ICS between age groups. (**B** and **C**) All T_H_2 (B) and T_H_1 (C) CD4^+^ T cell cytokines evaluated by MSD between age groups. (**D**) Eotaxin, IL-10, and IL-17A secretion detected by MSD between age groups. In (A), each dot represents an individual participant. The boxes with bold lines represent the geometric means and the 95% CIs for each group. In (B to D), each gray line represents an individual participant. Bold lines and shaded areas represent the geometric means and the 95% CIs for each group. In all panels, the *y* axis was log_10_ transformed.

These data collectively demonstrate that mRNA-1345 elicits a predominantly T_H_1-skewed CD4^+^ T cell response, with minimal induction of T_H_2 or other cytokines in older and younger adults—a favorable immune profile for protection against RSV.

### Polyfunctionality of Pre-F–specific CD4^+^ and CD8^+^ T cells is enhanced by mRNA-1345 vaccination across age groups and remained high at 2 years after vaccination

Polyfunctionality, defined as the ability of individual T cells to simultaneously produce or express multiple cytokines or activation markers, reflects the breadth of effector functions and acts as a key indicator of overall quality and functional fitness of T cell response (*58*–*64*).

To quantitatively assess polyfunctionality of Pre-F–specific T cell response elicited by mRNA-1345, we used the combinatorial polyfunctionality analysis of antigen-specific T cell subsets (COMPASS) (*65*) analysis on our ICS dataset. COMPASS is a Bayesian statistical model designed to analyze high-dimensional flow cytometry data by integrating both the number of antigen-responsive subsets and the number of functions expressed within each responsive subset, generating a polyfunctionality score (PFS) as a summary metric of T cell quality (*65*).

Using this approach, we found that baseline PFS values for both CD4^+^ and CD8^+^ T cells were relatively low across age groups (Fig. 4 and fig. S3). Following mRNA-1345 vaccination, PFS values for both CD4^+^ and CD8^+^ T cells were markedly elevated, indicating robust induction of high-quality T cell response with high functional fitness (Fig. 4 and fig. S3). Although a gradual contraction in PFS was observed over time, PFS for both CD4^+^ and CD8^+^ T cells remained substantially elevated above baseline even at 2 years after vaccination, highlighting this impact as durable (Fig. 4). Although the PFS was comparable between age groups at day 8, younger individuals exhibited higher PFS at intermediate time points such as days 15, 29, 91, 181, and 541. However, by 2 years after vaccination, CD4^+^ and CD8^+^ PFS values converged, showing no statistical differences between younger and older cohorts (Fig. 4).

**Fig. 4.**
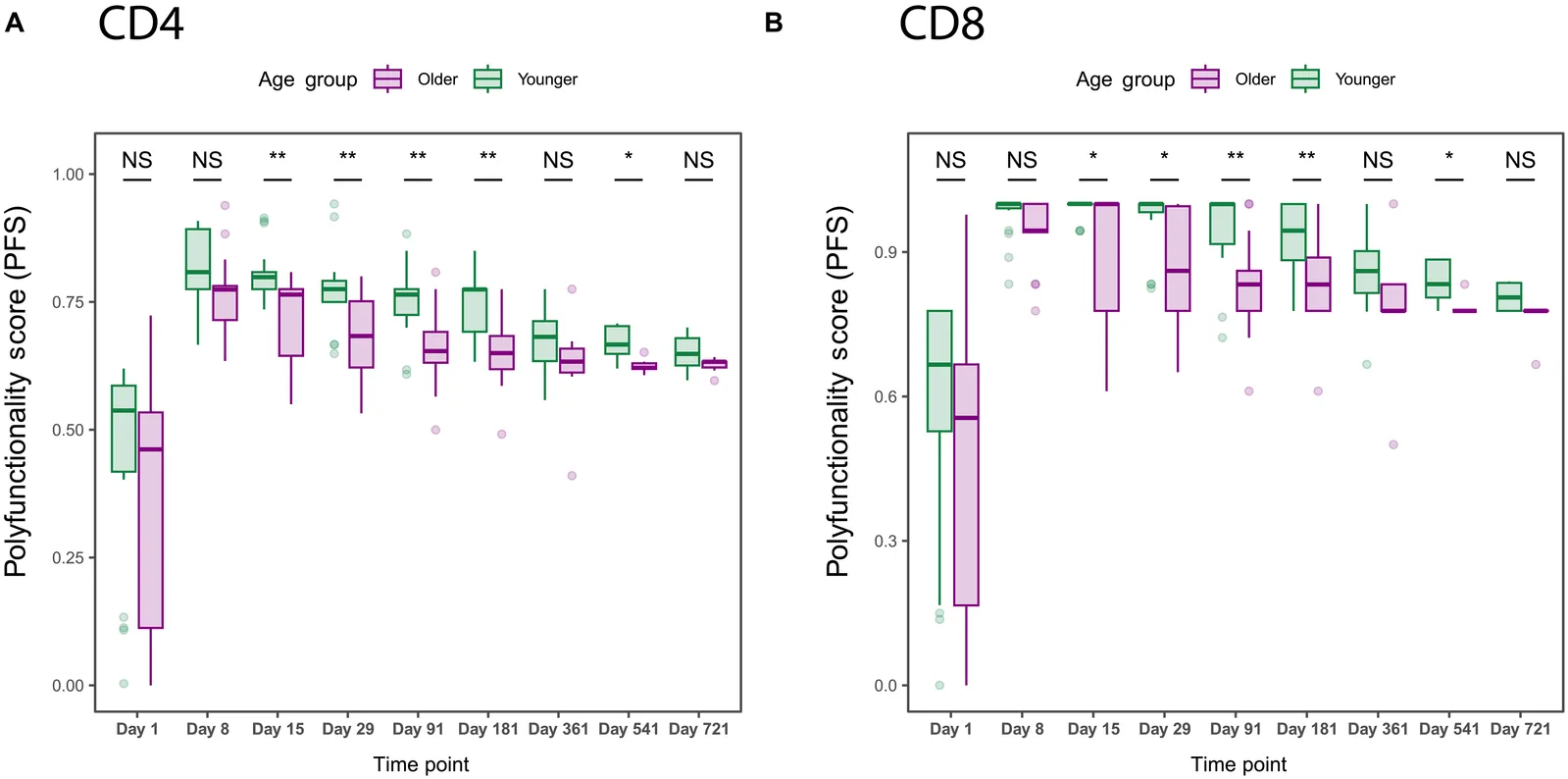
Polyfunctionality of Pre-F–specific CD4^+^ and CD8^+^ T cell responses is enhanced across age groups following mRNA-1345 vaccination and remained elevated at 2 years after vaccination. (**A** and **B**) PFS of CD4^+^ (A) and CD8^+^ (B) T cell response between age cohorts and at each time point by COMPASS analysis. Bold lines and boxes represent the medians and the central 50% of data [interquartile range (IQR)] for each group. The dots indicate outliers. A Mann-Whitney *U* test with Benjamini-Hochberg multiple correction was used to test for differences between age groups (*P < 0.05; **P < 0.005; ***P < 0.0005; ****P < 0.0001). NS, not significant.

These findings demonstrate that mRNA-1345 induces long-lasting, polyfunctional T cell response of high quality and fitness across age groups.

### Pre-F–specific CD4^+^ and CD8^+^ T cells exhibit comparable phenotypic and transcriptional profiles across age groups following mRNA-1345 vaccination

To investigate age-related differences in the phenotype and transcriptional states of RSV Pre-F specific T cells, we performed integrated single-cell RNA and TCR sequencing of CD4^+^ and CD8^+^ T cells isolated from PBMCs (Fig. 1). Given that day 15 PBMCs contained the highest frequency of antigen-specific (AIM^+^) T cells, cells were FACS sorted at this time point using AIM combinations, as described in Fig. 2 (E and F). In parallel, non-naïve T cells were isolated on the basis of exclusion of naïve T cells (CD45RA^+^CCR7^+^) during FACS. High-quality transcriptomic data were recovered, with ∼70% of cells containing paired TCRs, enabling robust tracking of clonotypes.

Uniform manifold approximation and projection (UMAP) of transcriptomic data revealed diverse CD4^+^ and CD8^+^ T cell subsets across both younger and older adults (Fig. 5, A and B, and fig. S4A). Marker gene expression confirmed canonical phenotypes, including effector, memory, cytotoxic, and terminal effector cells (T_emra_ cells) (Fig. 5, A and B). Subset distributions at baseline (day 1) and after vaccination (day 15 AIM^+^) were broadly similar between age groups (Fig. 5, C and D). Within the AIM^+^ CD8^+^ T cell population, effector memory cells (T_em_ cells) were predominant across all donors. Although a trend toward increased terminal effector CD8^+^ cells was noted in some older individuals, this pattern was driven primarily by a single donor (Donor 36) (Fig. 5D). In the AIM^+^ CD4^+^ compartment, activated CD4^+^ T cells were the dominant phenotype, followed by central memory subsets. A slight enrichment of activated CD4^+^ T cells was observed in older adults, but overall subset distributions remained comparable across age groups. Minor populations of naïve cells were also detected in both compartments, likely reflecting background contamination or sorting artifacts. Minimal levels of Foxp3^+^ regulatory T cells were also detected, likely due to their natural expression of CD25 and sometimes OX40. T_H_17-like CD4^+^ T cells were detected at very low frequencies and exhibited minimal IL-17A secretion, suggesting underrepresentation due to low transcript recovery (Figs. 3D and 5C).

**Fig. 5.**
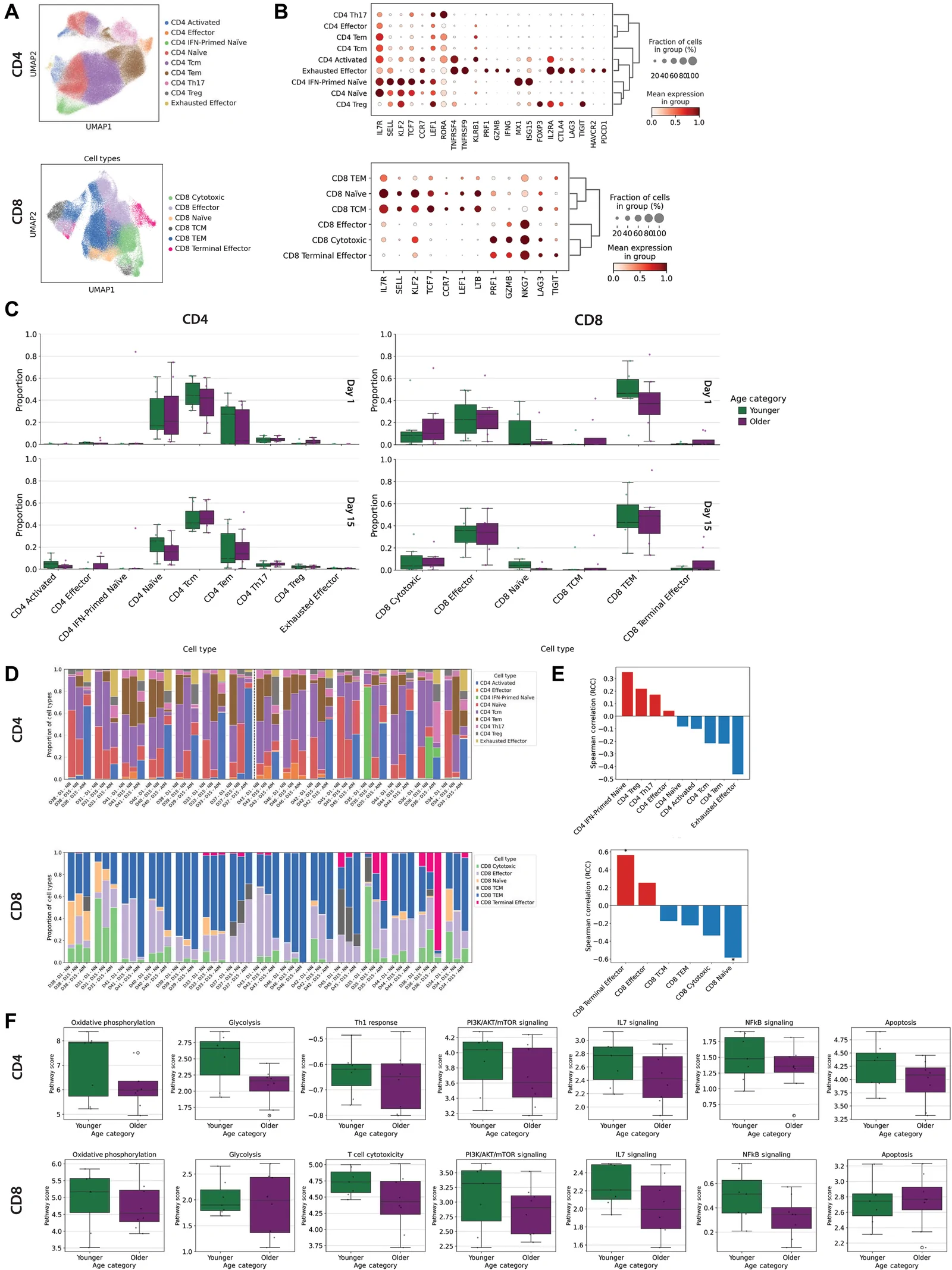
Global and Pre-F–specific CD4^+^ and CD8^+^ T cells exhibit comparable phenotypic composition and transcriptional profiles across age groups following mRNA-1345 vaccination. (**A**) UMAP visualization of single-cell transcriptomes from all donors. (**B**) Representative marker genes for T cell types. Dot size reflects the proportion of expressing cells; color intensity reflects average scaled expression. (**C**) T cell subset frequencies between age categories at day 1 and day 15. Bold lines and boxes represent the medians and the central 50% of data (IQR) for each group; whiskers extend to 1.5× IQR. (**D**) CD4^+^ and CD8^+^ T cell subset composition per donor at day 1 (D1), day 15 AIM^−^ (D15 NN), and day 15 AIM^+^ (D15 AIM^+^), ordered left to right by increasing age. (**E**) Spearman rank correlation coefficients (RCC) between donor age and the relative frequency of each T cell subtype among D15 AIM^+^ cells. (**F**) Gene signature scores of CD8^+^ T cells in younger and older adults. (A to F) *P < 0.05; **P < 0.005; ***P < 0.0005; ****P < 0.0001.

To further evaluate how antigen-specific T cell subset frequencies change with age, we performed a Spearman rank correlation analysis between donor age and the relative abundance of each day 15 AIM^+^ CD4^+^ and CD8^+^ T cell subsets across all individuals. Although several subsets showed nonsignificant trends, only the CD8^+^ T_emra_ cell population demonstrated a statistically significant positive correlation with age (Fig. 5E), indicating an age-associated enrichment of this subset in the antigen-specific CD8^+^ T cell pool that is consistent with the literature (*66*–*68*). No significant associations were observed among CD4^+^ T cell subsets.

We next assessed whether mRNA-1345 induces functional transcriptional differences in CD4^+^ and CD8^+^ T cells between younger and older adults. Differential gene expression analysis of AIM^+^ T cells revealed few age-associated changes beyond TCR-related transcripts (fig. S4B). To further explore functional distinctions, we performed targeted pathway enrichment analysis in Pre-F–specific CD8^+^ T cells (Fig. 5F). We observed a trend of lower expression of oxidative phosphorylation (OXPHOS) and glycolysis gene signatures in both CD4^+^ and CD8^+^ T cells from older adults, suggesting a shift in metabolic profile. A comparable effector gene expression (“T_H_1 response” pathway for CD4^+^ T cells and “T cell cytotoxicity” pathway for CD8^+^ T cells) was also observed in older adults, indicating mRNA-1345 induced T cells with comparable function in older adults, which is consistent with ICS data (Figs. 2 and 5F and fig. S2). In addition, we observed similar expression of IL-7, nuclear factor κB (NF-κB), phosphatidylinositol 3-kinase (PI3K)/AKT, and apoptosis gene pathways—all essential pathways for T cell survival and memory formation—indicating largely preserved long-term survival potential in both age groups (*69*–*77*) (Fig. 5F). Overall, these results suggest that the transcriptional landscape of mRNA-1345–induced T cells remains conserved across age groups.

### mRNA-1345–induced CD4^+^ and CD8^+^ T cells exhibit age-independent clonal dynamics with distinct patterns of expansion and repertoire overlap

To characterize the clonal architecture of the T cell response to mRNA-1345, we analyzed global TCR repertoires of CD4^+^ and CD8^+^ T cells. UMAP of clonal family size revealed limited clonal expansion among CD4^+^ T cells, with most clonotypes represented by single cells and few expanded beyond small clusters (Fig. 6, A to C, and fig. S5, A and B). This broadly polyclonal profile was reflected in consistently high D50 values (all >40), indicating that a large number of clonotypes contribute evenly to the CD4^+^ repertoire across both younger and older individuals (Fig. 6D).

**Fig. 6.**
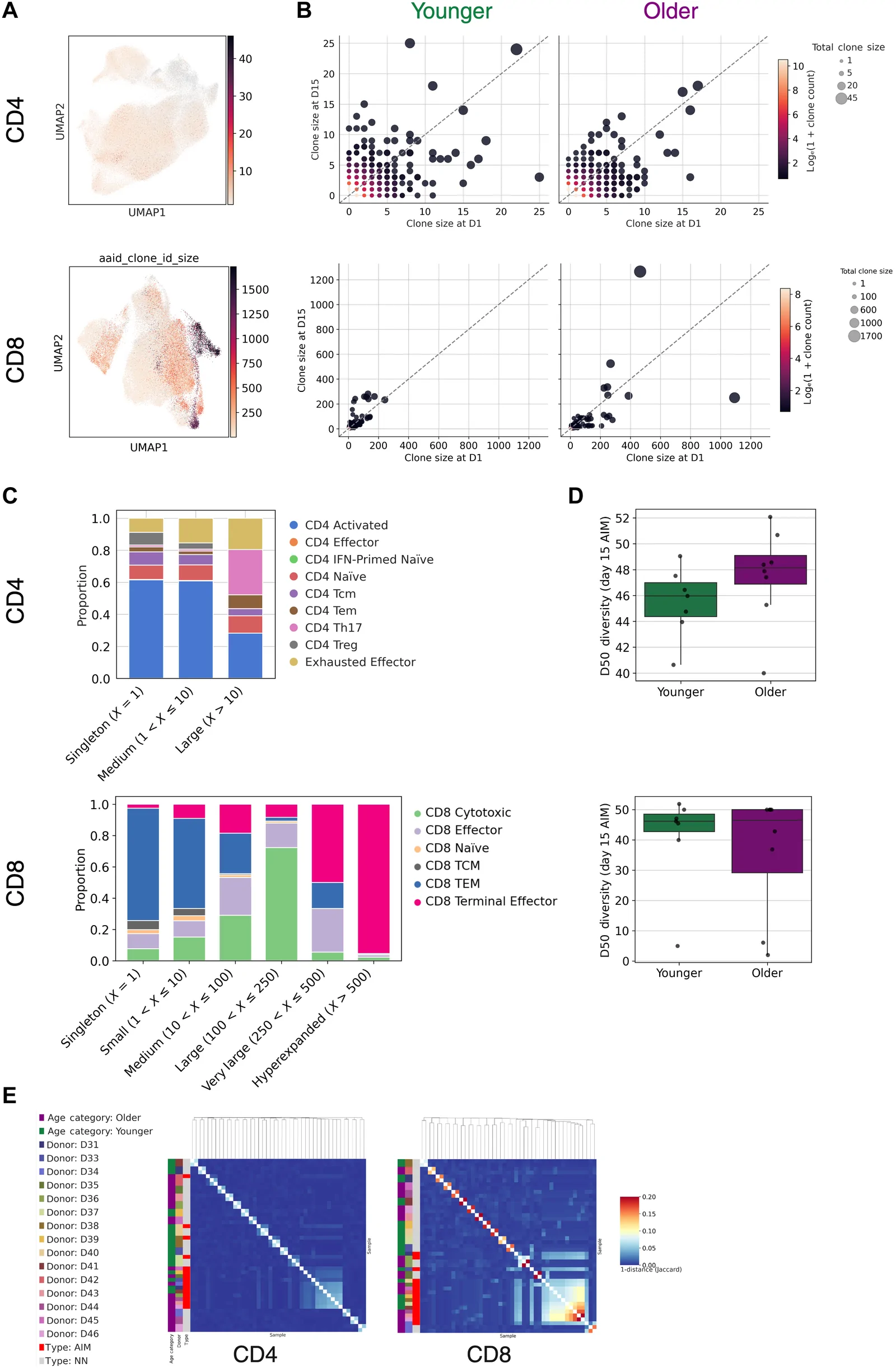
mRNA-1345–induced CD4^+^ and CD8^+^ T cells exhibit clonal overlap across age groups with similar CD4^+^ clonotype kinetics. (**A**) UMAPs showing the clonal expansion of T cells based on amino acid sequence identity. Each point represents a single cell, colored by the size of its clonal family. Color scales indicate the number of cells per clonotype on a log-transformed scale. (**B**) Scatterplots showing the clone size at day 15 versus day 1 for CD4^+^ (top) and CD8^+^ (bottom) T cells, stratified by age group. Each point represents a unique clonotype, with dot size indicating the total number of cells in that clone across both time points, and color intensity reflecting the number of clonotypes sharing a given D1,D15 coordinate [log_e_(1 + clone count)]. Dashed lines indicate the 1:1 diagonal. (**C**) Composition of CD4^+^ (top) and CD8^+^ (bottom) T cell subsets stratified by clonal expansion. Each bar represents the proportion of annotated cell types within each clonal size bin. (**D**) D50 index of day 15 AIM^+^ CD4^+^ (top) and CD8^+^ (bottom) T cells. Each dot represents an individual participant. Boxes with the center lines represent the geometric means and the 95% CIs. (**E**) Heatmaps showing pairwise CD4^+^ and CD8^+^ TCR repertoire similarity between samples, based on the amino acid alignment of CDR3β sequences. Warmer colors indicate greater overlap.

In contrast, CD8^+^ T cells exhibited more pronounced oligoclonal expansion, with several clonotypes reaching sizes exceeding 1500 cells (Fig. 6, A to C, and fig. S5, A and B). The D50 values for CD8^+^ T cells were correspondingly lower and more variable in both age groups, ranging from 1 to ∼40, suggesting that, in some individuals, a small number of dominant clones accounted for a majority of the response (Fig. 6D). This clonal dominance was not age dependent as individuals with low D50 profiles were observed in both younger and older donors (Fig. 6D).

Subset composition analysis further supported these differences. CD4^+^ T cells showed relatively uniform representation of functional subsets across clonal sizes, with a modest enrichment of T_H_17 cells among the largest clonotypes (Fig. 6C). In contrast, the most highly expanded CD8^+^ clones were overwhelmingly composed of T_em_ cells (Fig. 6C).

In addition to the D50 index (Fig. 6D), we used Shannon entropy (fig. S5C) to access the clonal diversity of antigen-specific CD4^+^ and CD8^+^ T cell response between age groups. The D50 index quantifies the minimal number of unique clonal types to account for 50% of the total TCR repertoire, and the Shannon entropy reflects both richness and evenness of the repertoire. Using both metrics provided us with a more comprehensive understanding of the clonal architecture, capturing shifts in either clonal dominance or clonal distribution that might have been overlooked by a single metric. Using these complimentary approaches, we observed comparable clonal diversity of CD4^+^ and CD8^+^ T cell response between younger and older adults (Fig. 6D and fig. S5C). Collectively, these findings indicate that aging does not markedly alter the breadth or evenness of the vaccine-induced T cell repertoire following mRNA-1345 vaccination.

Next, we assessed interindividual TCR repertoire similarity by clustering CDR3β amino acid sequences based on pairwise sequence similarity. AIM^+^ repertoires, particularly within the CD8^+^ compartment, were grouped together across multiple donors and age categories (Fig. 6E), indicating shared TCR motifs in response to immunodominant RSV epitopes. In the CD4^+^ compartment (Fig. 6E), we also observed clustering of AIM^+^ repertoires across donors, but the overlap was less pronounced compared with CD8^+^ T cells. This suggests a moderate degree of TCR convergence in CD4^+^ responses, possibly reflecting recognition of shared epitopes but with greater repertoire diversity, consistent with broader major histocompatibility complex II–restricted antigen presentation.

Together, these data show that mRNA-1345 induces robust CD4^+^ T cell priming across age groups, with a diverse polyclonal repertoire. In contrast, CD8^+^ responses are more oligoclonal, driven largely by effector and T_em_ cell phenotypes. Repertoire overlap analysis reveals strong convergence of CD8^+^ AIM^+^ clonotypes across individuals, suggesting targeting of shared epitopes, whereas CD4^+^ repertoires are more diverse, with moderate interindividual similarity.

## DISCUSSION

The immune response to RSV is highly complex (*10*–*19*). Although neutralizing antibodies are key for limiting viral entry and reducing acute viral load (*20*–*23*), T cells—particularly CD8^+^ CTLs and T_H_1 CD4^+^ subsets—are essential for viral clearance and disease resolution (*24*, *27*–*32*). Here, we demonstrate that mRNA-1345 induces robust, polyfunctional, CD8^+^ cytotoxic T cell response and T_H_1-polarized CD4^+^ T cell response in both younger and older adults, with CD4^+^ T cell response sustained at 2 years after vaccination. Our comprehensive profiling reveals that mRNA-1345–elicited T cell responses in older individuals are largely comparable to those observed in younger adults in magnitude, kinetics, quality, and persistence. These findings are especially relevant in the context of RSV vaccine development, as they support the potential of mRNA-1345 to close a critical immunity gap in high-risk aging populations. Immunosenescence is likely a key contributor to this increased susceptibility, potentially through delayed or suboptimal T cell responses that impair early viral control. Similar associations have been observed in the context of COVID-19, where delayed T cell kinetics correlate with worse clinical outcomes (*78*–*83*). These parallels underscore the importance of developing vaccines that can overcome age-related immune dysfunction and effectively elicit T cell–mediated protection in older populations.

Aging affects nearly all facets of the T cell response, including delayed response kinetics, diminished proliferative capacity, impaired cytokine production, and reduced cytotoxic function (*34*–*37*). This presents a major challenge for RSV vaccine development, as older adults are at heightened risk for severe RSV disease (*7*). To assess the impact of age on mRNA-1345 induced immunity, we evaluated the T cell response between older and younger adults both quantitatively and qualitatively with in-depth profiling of cell status.

In our study, we observed that neither CD4^+^ nor CD8^+^ T cell responses were affected by aging, with similar frequencies observed at peak immunity. Furthermore, vaccine-induced CD4^+^ T cell response was maintained for up to 2 years after vaccination. Single-cell transcriptomic analysis further showed that mRNA-1345–elicited T cells retained high levels of polyfunctionality in older adults, with minimal transcriptional hallmarks of dysfunction. Most differentially expressed genes were related to TCR identity, likely reflecting the pronounced clonal expansion observed within the CD8^+^ T cell compartment. The PI3K/AKT pathway, a key regulator of T cell survival and memory formation, showed comparable expression across age groups, suggesting that mRNA-1345–induced T cells retain the potential for long-term persistence (*69*, *70*). Notably, effector gene expression was conserved in older adults, which is consistent with our ICS data. These findings indicate that mRNA-1345 can elicit high-quality cellular immunity in older individuals, effectively overcoming traditional barriers posed by immunosenescence (*58*–*64*).

Aging is also accompanied by shifts in global immune cell composition and alterations in cellular metabolic profiles (*68*, *84*–*91*), both of which can influence vaccine responsiveness. In our study, T cell subset distributions were broadly similar between younger and older adults at both baseline and postvaccination time points. AIM^+^ cells were predominantly composed of T_em_ CD8^+^ T cells and were consistently represented across donors. Although T_emra_ cells were highly expanded in a subset of older individuals and derived from preexisting clones, this trend was not consistent across the cohort. In AIM^+^ CD4^+^ T cells, activated and central memory phenotypes were dominant and broadly comparable between age groups. At the transcriptional level, we detected no difference in the OXPHOS pathway in AIM^+^ CD4^+^ and CD8^+^ T cells from older individuals. This finding suggests mRNA-1345–elicited CD4^+^ and CD8^+^ T cells do not display defective OXPHOS, which is commonly reported in aged memory and effector T cells (*92*–*94*). We also did not observe the commonly reported down-regulation of glycolytic pathways (*92*–*94*) in AIM^+^ CD4^+^ T cells from older individuals. The conserved metabolic capacity may contribute to the preservation of T cell function and longevity in older individuals in response to mRNA-1345 vaccination.

The mRNA lipid nanoparticle (LNP) vaccine platform offers several advantages over conventional platforms, including intrinsic adjuvanting effects, balanced humoral and cellular immunity, compatibility with antigen multivalency, and versatility (*95*–*97*). mRNA LNP–based vaccines are well documented as eliciting strong CD4^+^ T cell response with a predominant T_H_1 profile (*98*–*103*)—a particularly desirable feature for RSV vaccine development, given the association of T_H_2- or T_H_17-skewed response with severe RSV disease (*51*–*53*). In line with this, we observed a robust T_H_1-biased CD4^+^ T cell response following mRNA-1345 vaccination, with minimal evidence of T_H_2 polarization. Our ICS data revealed only minimal IL-4– or IL-5–producing CD4^+^ T cells, whereas MSD analysis showed minimal levels of IL-5 but no detectable IL-4 in culture supernatant. These minor discrepancies likely reflect the methodological differences between the assays, including variations in incubation time and analyte stability during incubation and cryo-storage. Nonetheless, both ICS and MSD data consistently demonstrate that the Pre-F–specific CD4^+^ T cell response is T_H_1 polarized in nature, which aligns with a favorable immunologic signature for RSV protection and further supports the potential of mRNA-1345 in eliciting protective cellular immunity in older adults.

To comprehensively assess the function and quality of vaccine-induced T cells, we used COMPASS analysis, which quantifies both the breadth and depth of polyfunctional responses. This approach captured nuanced differences in cytokine coexpression patterns not readily detectable by traditional ICS analysis, which primarily quantifies the frequencies of predefined T cell subsets. This approach provides an extensive measurement of T cell polyfunctionality, which has been associated with improved immune protection in various settings (*58*–*64*). By leveraging COMPASS analysis, we were able to capture subtle yet meaningful differences in T cell functional profile that may be overlooked by traditional flow cytometry–based analysis. For example, at day 721 after vaccination, COMPASS identified a higher PFS for the Pre-F–specific CD8^+^ T cells compared with baseline. Although the frequency of these cells is very low, the analysis revealed difference in polyfunctionality, suggesting that, at low frequency, the quality of Pre-F–specific CD8^+^ T cells at day 721 is improved compared with baseline. However, COMPASS analysis also has limitations, including its reduced sensitivity in low-frequency populations (*65*). In addition, the interpretation of COMPASS-derived PFSs can be dependent on the number of functions measured, meaning scores cannot be compared between CD4^+^ (four functions) and CD8^+^ (three functions) T cells. Despite these limitations, COMPASS analysis offers a powerful tool to dissect the functional landscape of Pre-F–specific T cells and provides further evidence of mRNA-1345 eliciting robust T cell response with high functional fitness in older adults.

It has been reported that aging is associated with narrowing of the TCR repertoire due to thymic involution and cumulative antigen exposure, which can lead to reduced clonal diversity in older individuals (*34*–*37*). To evaluate whether mRNA-1345–induced responses are affected by this age-associated constraint, we analyzed TCR repertoire architecture using the D50 index. We found that CD4^+^ T cells exhibited a broadly polyclonal distribution across both age groups, with high D50 values and limited clonal expansion. Notably, we did not observe an age-related decline in CD4^+^ T cell diversity in response to mRNA-1345, suggesting that the vaccine is capable of inducing a sufficiently broad TCR response even in older individuals. CD8^+^ T cells, by contrast, showed more pronounced oligoclonal expansion, consistent with known patterns of cytotoxic T cell responses. Although some individuals exhibited lower D50 values for their CD8^+^ T cell response, this was observed in both younger and older individuals and reflected interindividual variability rather than age-specific repertoire contraction. It is important to note that diversity metrics such as D50 are sensitive to sequencing depth and may underrepresent rare clones, whereas other indices can be influenced by sample size and clonal expansion kinetics (*104*–*108*).

Furthermore, repertoire overlap analysis revealed notable convergence of Pre-F–specific CD4^+^ and CD8^+^ T cell clonotypes across donors, regardless of age. This suggests that mRNA-1345 induces public TCR responses to shared immunodominant epitopes. These patterns of clonal overlap align with comparable magnitudes and diversity of cytokine-producing Pre-F–specific CD4^+^ T cells across age groups (Figs. 2 and 6). Together, these data highlight the ability of mRNA-1345 to induce consistent and effective CD4^+^ and CD8^+^ T cell responses irrespective of age, reinforcing its potential for broad protective efficacy.

One limitation of our study is the small sample size in the multiomic dataset, which included only seven younger and eight older subjects. Although the results are promising, the limited cohort size restricts statistical power and generalizability. Future studies should incorporate larger, more age-stratified cohorts—including individuals over 70—and extend analysis to later time points after vaccination. This will enable a more comprehensive understanding of long-term T cell responses and help define correlates of protection in older adults.

Despite the induction of robust CD4^+^ and CD8^+^ T cell response and the persistence of durable T_H_1-polarized CD4^+^ cells, the observed vaccine efficacy of RSV vaccines—measured by protection against lower respiratory tract disease—waned 2 years after vaccination. This decrease in protection could be primarily attributed to declining neutralizing antibody titers, which play a central role in preventing viral entry and limiting early replication in the respiratory tract (*20*–*23*). Although humoral immunity is often the primary correlate of protection against infection, cell-mediated immunity—particularly T_H_1-polarized CD4^+^ T cell responses—may contribute to protection through alternative mechanisms, including supporting CD8^+^ T cell cytotoxic function, enhancing the quality and magnitude of recall response, and facilitating viral clearance by promoting innate immune activation (*24*–*32*). T_H_1-polarized CD4^+^ T cells may help reduce immunopathology by regulating inflammatory response during RSV infection. Hence, although the drop in neutralizing antibody titers may allow for breakthrough infections, the persistence of a potent T_H_1-polarized CD4^+^ T cell response could still provide clinically meaningful benefit, such as reducing the severity of disease, minimizing tissue damage in lungs, and accelerating recovery (*109*). The comprehensive analyses performed in this study identify the potential mechanisms whereby mRNA vaccines induce durable, polyfunctional T cell responses in both younger and older adult populations. Future studies, particularly in settings where neutralizing antibody levels have waned, may further shed light on the potential role of durable T_H_1-polarized CD4^+^ T cell response in shaping long-term vaccine-mediated protection, even as humoral immunity declines.

## MATERIALS AND METHODS

### Study design

This study is a part of the open-label, randomized, two-part, phase 1 clinical trial (NCT05397223) evaluating the safety, reactogenicity, and immunogenicity of several of Moderna’s mRNA-LNP vaccines, including mRNA-1345. The study protocol was reviewed and approved by the Advarra Institutional Review Board (IRB; protocol no. Pro00061954). As previously described (*101*), the mRNA-1345 arm enrolled participants in two age groups, younger adults (aged 18 to 49) and older adults (aged 50 to 75). Twenty-nine younger adults and 32 older adults, who were in good health and had no recent vaccination or infection with RSV, received a single dose of mRNA-1345 50 μg on day 1. A subset of participants was randomly selected for the T cell analysis listed below. Comprehensive participant demographics, eligibility criteria, and study procedures for this study cohort have been reported previously (*101*).

Workstream 1: Days 1, 8, 15, 29, 91, 181, 361, 541, and 721 PBMC samples were subjected to stimulation with the Pre-F peptide pool followed by ICS analysis to assess the magnitude of RSV Pre-F–specific T cell response. Nineteen younger adults and 17 older adults were included.

Workstream 2: Days 1, 8, 15, 29, and 91 PBMC samples were subjected to stimulation with the Pre-F peptide pool. The cells were used for AIM analysis, whereas the culture supernatant was analyzed by 10-plex MSD assay for secreted cytokines. Twenty-seven younger adults and 28 older adults were included.

Workstream 3: Day 15 PBMC samples were subjected to FACS based on AIM positivity followed by coupled single-cell RNA and TCR sequencing. Seven younger adults and 8 older adults were included.

### Study vaccines and procedures

mRNA-1345 50 μg is an RSV vaccine encompassing a single mRNA encoding a membrane-anchored RSV-F glycoprotein, derived from an RSV-A strain, and stabilized in the prefusion conformation (*110*). mRNA-1345 was administered as an intramuscular injection (0.5 ml), and all participants were followed for ≥12 months after the last vaccine dose as previously described (*101*).

### Blood samples

Blood samples were collected from healthy individuals vaccinated with mRNA-1345 at baseline (day 1), 7 days (day 8), 2 weeks (day 15), 1 month (day 29), 3 months (day 91), 6 months (day 181), 12 months (day 361), 18 months (day 541), and 24 months (day 721) after vaccination as a part of the mRNA-CRID-001 clinical trial (clinicaltrials.gov identifier NCT05397223). PBMC samples were prepared from whole blood specimens using standard Ficoll separation methods and were cryopreserved and stored in liquid nitrogen vapor phase until testing. Details on the study design and participants, study objectives and endpoints, study vaccine and procedures, and humoral immunogenicity data up to day 29 have been previously reported (*101*). The protocol was approved by a central institutional review board. The study was conducted in accordance with the protocol, the principles of the International Council for Harmonisation Good Clinical Practice guidelines, the Declaration of Helsinki, and national, state, and local laws/regulations. All participants provided written informed consent for participation in the study, including all evaluations and study procedures specified by the protocol.

### AIM and MSD assay

PBMCs were thawed and rested overnight before being stimulated with the RSV Pre-F peptide pool (GenScript, customized pool containing mutations that stabilize the F protein in the Pre-F conformation; 2 μg/ml), mitogens, or dimethyl sulfoxide (DMSO) for 22 to 24 hours. The next day, the cell culture supernatant was collected for all stimulation conditions and analyzed with a customized 10-plex MSD U-plex assay per the manufacturer’s protocol. Surface staining was performed using a panel of fluorescently labeled antibodies targeting various T cell markers, including OX40, CD25, CD69, and CD137. A full list of mutations in the Pre-F peptide pool [15–amino acid peptides with 11–amino acid overlaps between sequential peptides) compared to wild-type (WT) pool is shown in table S1. A full list of antibodies used for AIM assay can be found in table S3. Data acquisition was performed on Cytek Aurora flow cytometers, and spectral unmixing was conducted using the SpectroFlo software. Gating and Boolean analysis were performed on the OMIQ.

### ICS assay

PBMCs were thawed and rested overnight before being stimulated with peptide pools (GenScript, customized pool containing mutations that stabilize the F protein in the Pre-F conformation; 2 μg/ml), mitogens, or DMSO in the presence of protein transport inhibitors for 7.5 hours. Surface and intracellular staining were performed using a panel of fluorescently labeled antibodies targeting various T cell markers and cytokines. Functional markers included IFN-γ, TNF, IL-2, IL-4, IL-5, IL-13, IL-17A, CD40L, CD107a, and granzyme B. A full list of mutations in the Pre-F peptide pool (15–amino acid peptides with 11–amino acid overlaps between sequential peptides) compared to the WT pool is shown in table S1. A full list of antibodies used in ICS assay can be found in table S2. Data acquisition was performed on Cytek Aurora flow cytometers, and spectral unmixing was conducted using the SpectroFlo software. Gating and Boolean analysis were performed using the FCSExpress IVD version 7 or OMIQ.

### Single-cell RNA sequencing

Cell number and viability of single-cell suspensions were analyzed using a Thermo Fisher Scientific Countess III. Single-cell suspensions were loaded onto a Chromium Single Cell chip (10x Genomics) according to the manufacturer’s instructions for coencapsulation with barcoded gel beads at a target capture rate of ∼5000 individual cells per sample. Briefly, for each donor, non-naive and AIM^+^ T cells were sorted as described above and processed for single-cell sequencing using a Chromium Next GEM Single Cell 5′ Library & Gel Bead Kit v2 (10x Genomics, catalog no. PN-1000263), a Chromium Next GEM Chip K Single Cell Kit (10x Genomics, catalog no. PN-1000286), and a Chromium Controller (10x Genomics). All samples for a given donor were processed simultaneously with the Chromium Controller, and the resulting libraries were prepared in parallel. The gene expression and the fragmented TCR VDJ targeted enrichment libraries were prepared using a Chromium Single Cell 5′ Library Construction Kit (10x Genomics, catalog no. PN-1000020) and a Chromium Single Cell V(D)J Enrichment Kit (10x Genomics, catalog no. PN-1000005). All libraries were indexed using primers from the Dual Index Kit TT Set A (10x Genomics, catalog no. PN-1000215) and sequenced by next-generation analytics using an Illumina Novaseq (Illumina) and a single lane of an S4 flow cell.

### Single-cell gene expression analysis

The raw single-cell RNA sequencing (scRNA-seq) data were aligned to the human genome (GRCh38) using Cell Ranger (10x Genomics, v7.0.0). Downstream analysis was carried out using the Python package Scanpy (version 1.10.0).

Cells were preprocessed using BESCA (version 2.2.5) to remove low-quality or degraded cells, and Scrublet was used to identify and exclude doublets. Individual cells were retained on the basis of the following criteria: (i) detection of more than 200 genes, (ii) less than 10% of reads mapped to mitochondrial genes, and (iii) expression of genes in more than 10 cells.

To improve the resolution of T cell annotation, the dataset was split into CD4^+^ and CD8^+^ T cells for more targeted subtyping. Leiden clustering was then performed within Scanpy to group cells into distinct clusters. Cluster-specific marker genes were identified using the rank_genes_groups function using the Wilcoxon rank sum test. These clusters were manually annotated for T cell subtypes using canonical marker genes.

### Paired single-cell TCR analysis

Raw single-cell TCR sequencing data were aligned to the VDJ-GRCh38-alts-ensembl (version 7.0.0) using Cell Ranger. The single-cell TCR sequencing data were then integrated with the scRNA-seq data based on unique cell barcodes, allowing for the pairing of transcriptomic and TCR sequence information for each individual cell. From this integration, we identified TCR α and β chain sequences for 224,634 of the 315,568 (71%) T cells present in the scRNA-seq dataset.

For downstream analysis, only cells with both a productive α chain and a productive β chain were retained. Subsequent analyses were performed using the Python package Scirpy (version 0.12.0). TCR clonotypes were defined using Scirpy’s pp.ir_dist and tl.define_clonotype_clusters functions based on amino acid sequence identity of the full TCR sequence, including both the α and β chains using receptor_arms=“all.” Clonotypes were then grouped into clonal categories based on the number of T cells expressing the same TCR clone.

### Clonal diversity analysis

Clonal diversity was quantified using Shannon entropy and the D50 index, which represents the proportion of unique clonotypes that account for 50% of the total repertoire. Diversity metrics were computed using the tl.alpha_diversity function in Scirpy. The aaid_clone_id field was used to define clonal identity.

### CDR3β clustering analysis

For the evaluation of TCR repertoire overlap across donor samples, the analysis was modified to specifically focus on β chain similarity. The tl.define_clonotype_clusters function was used, restricting the analysis to the β chain (receptor_arms=“VDJ”) and using sequence alignment (metric=“alignment”) to define clonotype clusters based on β chain similarity. This allowed for a more targeted analysis of β chain diversity across different donor samples.

To evaluate the similarity of TCR repertoires across donor samples, a matrix was constructed to capture the abundance of clonotypes in each sample. This matrix was generated using Scirpy’s tl.repertoire_overlap function to assess β chain similarity between repertoires.

### Transcriptional signature analysis

To assess the expression of signatures, we compared donor level medians of gene expression with the Wilcoxon test. Gene sets were obtained from the Molecular Signatures Database (MSigDB), including curated pathways from the KEGG, BioCarta, and GO Biological Processes collections. These gene sets were used for downstream enrichment and pathway-level analyses.

### COMPASS analysis of T cell PFSs

To evaluate antigen-specific T cell polyfunctionality, we applied COMPASS (*65*), a Bayesian hierarchical mixture model that identifies antigen-specific responses across all measurable T cell subsets simultaneously. The model uses cell count data from ICS assays under both unstimulated (DMSO) and antigen-stimulated conditions. For T cell functionality, we considered the expression of four cytokines (IFN-γ, TNF-α, IL-2, and CD40L) in CD4^+^ T cells, and three cytokines (IFN-γ, TNF-α, and IL-2) in CD8^+^ T cells. This resulted in 16 Boolean-defined functional subsets for CD4^+^ T cells and 8 for CD8^+^ T cells. COMPASS calculates the posterior probability of antigen-specific response for each subset per subject. These probabilities are then used to derive a PFS that summarizes the breath and functional capacity of the subject’s antigen-specific T cell response across all subsets.

### Statistical analysis

There was no formal hypothesis testing. All analyses and data summaries/displays were performed by age group and for the total population (where applicable) unless otherwise specified; data are presented as categorical variables, frequencies, and percentages. The geometric mean of T cell response by ICS, AIM, or MSD with corresponding 95% confidence intervals (CIs) at each time point and the geometric mean fold rise of specific analyte with corresponding 95% CIs at each postbaseline time point compared with prevaccination baseline at day 1 were calculated by age cohort. Percentage responders was defined as the proportion of participants who demonstrated responses ≥2-fold over baseline or those who had responses below LLOD at baseline but mounted responses ≥LLOD following vaccination. A Mann-Whitney *U* test with Benjamini-Hochberg multiple correction was used to test for differences in PFS between age groups. The Wilcoxon rank sum test with Holm-Bonferroni correction was used to compare the magnitude of IFN-γ^+^ CD4^+^ and CD8^+^ to assess age-related changes in T cell subsets. Spearman rank correlation was used to evaluate associations between cell type proportions and donor age. Mann-Whitney *U* tests were also performed to compare proportions between younger and older age groups; we calculated the proportion of each cell type per donor using single-cell RNA-seq data pooled across day 1 and day 15 time points. Adjusted *P* values with Benjamini-Hochberg multiple testing correction were used to control the false discovery rate (FDR) at a 5% threshold.
