# Supplementary material for: The mRNA-based RSV vaccine mRNA-1345 induces robust and durable CD4+ T cell responses against RSV in younger and older adults
Source: Sci Adv. 2026 Oct 21;12(43):eaeh4436. doi: 10.1126/sciadv.aeh4436 (PMC13685949; doi:10.1126/sciadv.aeh4436)
Supplement: Supplementary file 1 [file sciadv.aeh4436_sm.pdf]

Science Advances
AAAS

Supplementary Materials for

The mRNA-based RSV vaccine mRNA-1345 induces robust and durable $CD4^+$ T cell responses against RSV in younger and older adults

Anthony DiPiazza *et al.*

Corresponding author: Jaap Oostendorp, jaap.oostendorp@modernatx.com

Sci. Adv. **12**, eaeh4436 (2026)
DOI: 10.1126/sciadv.aeh4436

This PDF file includes:

Figs. S1 to S5
Tables S1 to S3

Fig. S1. Representative gating tree for ICS assay.

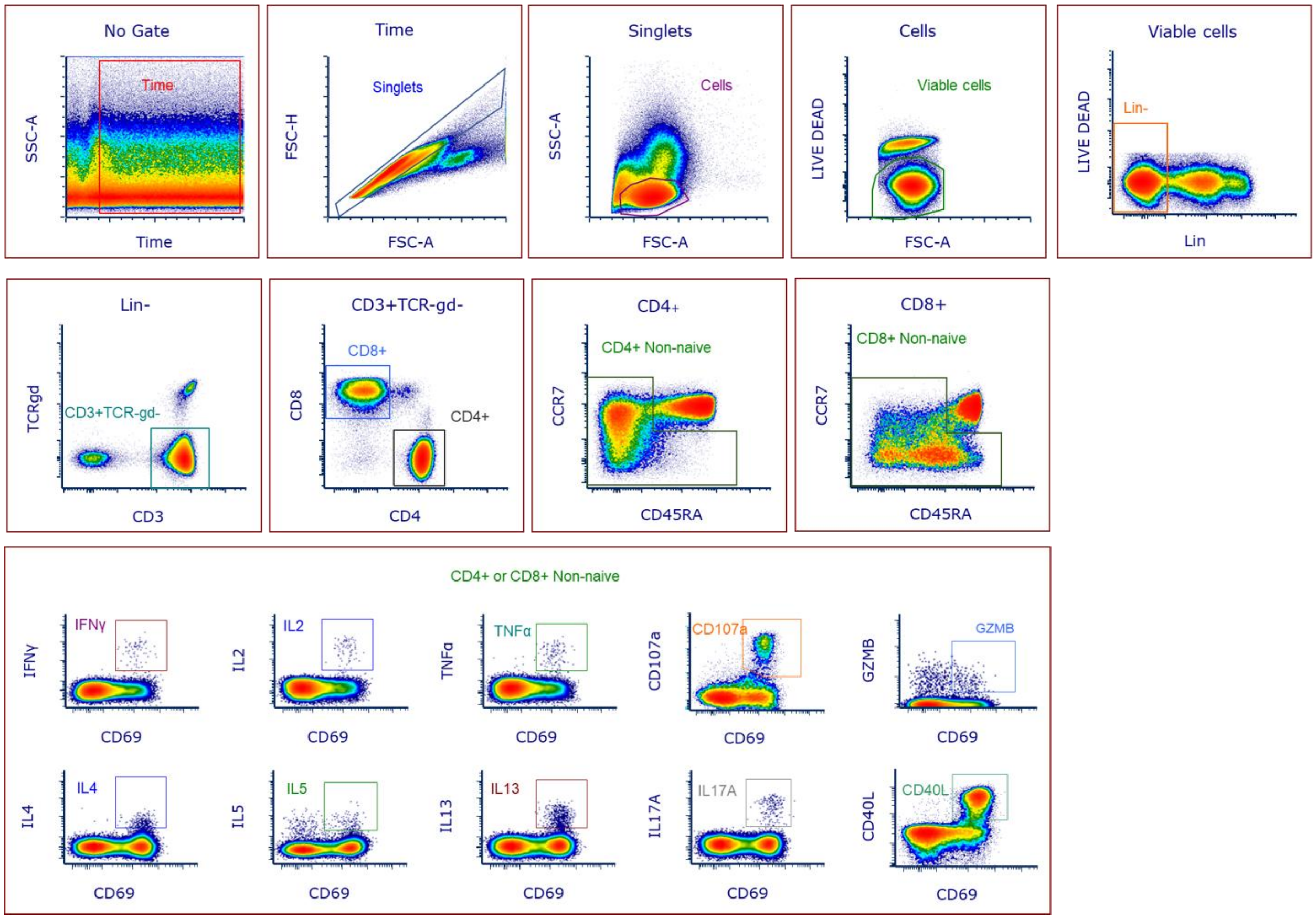

Fig. S2. mRNA-1345 elicits comparable CD4+ and CD8$^+$ T cell response across age groups throughout the study duration.

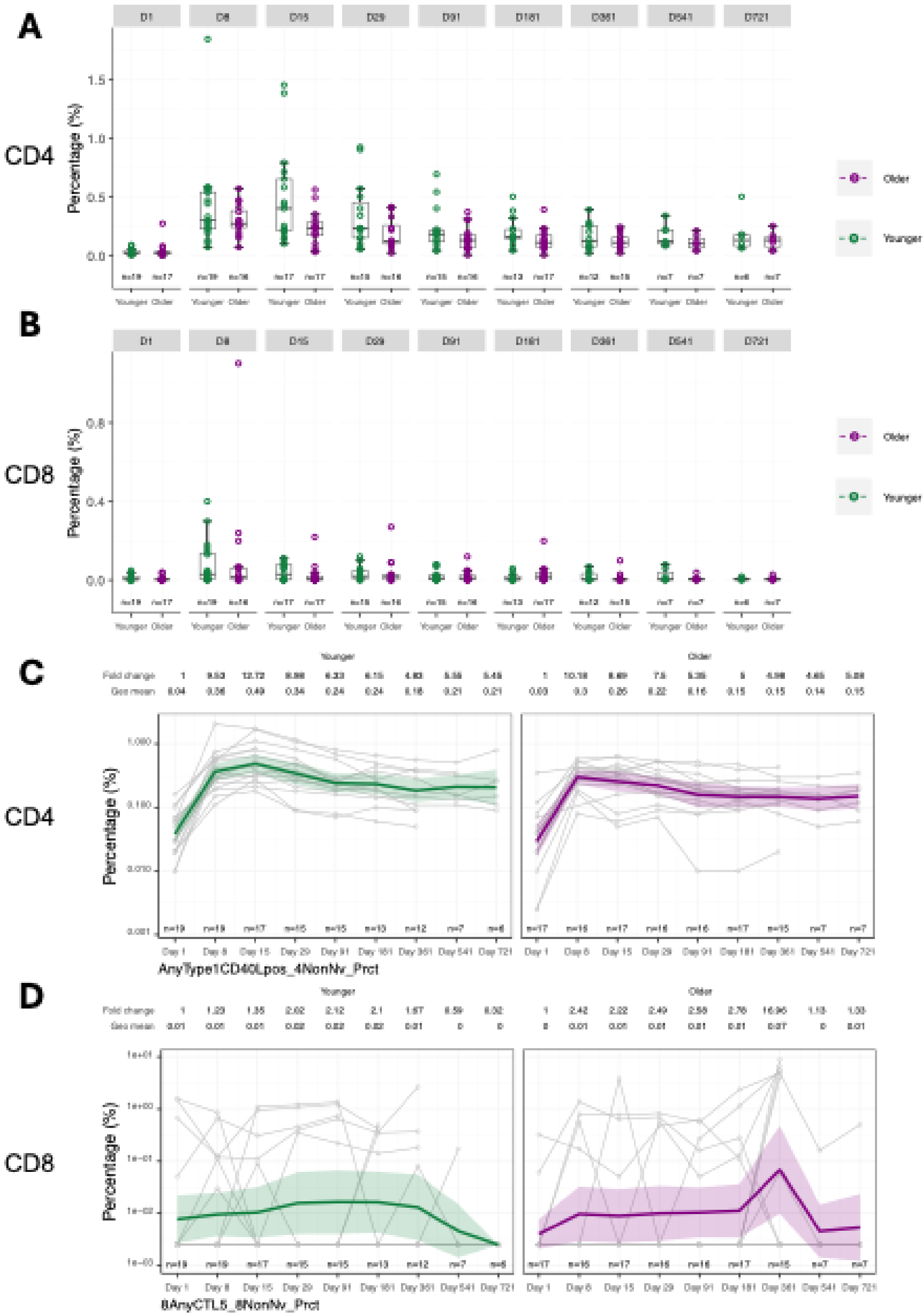

(**A**) IFN-γ^+ $CD4^+$ T cell response between age groups by ICS.

(**B**) IFN-γ^+ $CD8^+$ T cell response between age groups by ICS.

(**C**) Any Th1 $CD4^+$ T cell response between age groups by ICS. Any Th1 $CD4^+$ T cell responses were defined by expression of at least one of the following markers: IFN-γ, TNF-α, IL-2, and CD40L.

(**D**) Any CTL $CD8^+$ T cell response between age groups by ICS. Any CTL $CD8^+$ T cell responses were defined by expression of at least one of the following markers: IFN-γ, TNF-α, IL-2, and CD107a.

In panels (**A**) and (**B**), each dot represents an individual participant. Boxes with bold lines represent the geometric means and the 95% confidence intervals for each group.

In panels (**C**) and (**D**), each gray line represents an individual participant. Bold lines and shaded areas represent the geometric means and the 95% confidence intervals for each group. The y-axis was $\log_{10}$ transformed.

Fig. S3. Heatmaps of COMPASS posterior probabilities for antigen-specific CD4⁺ and CD8⁺ T cell subsets.

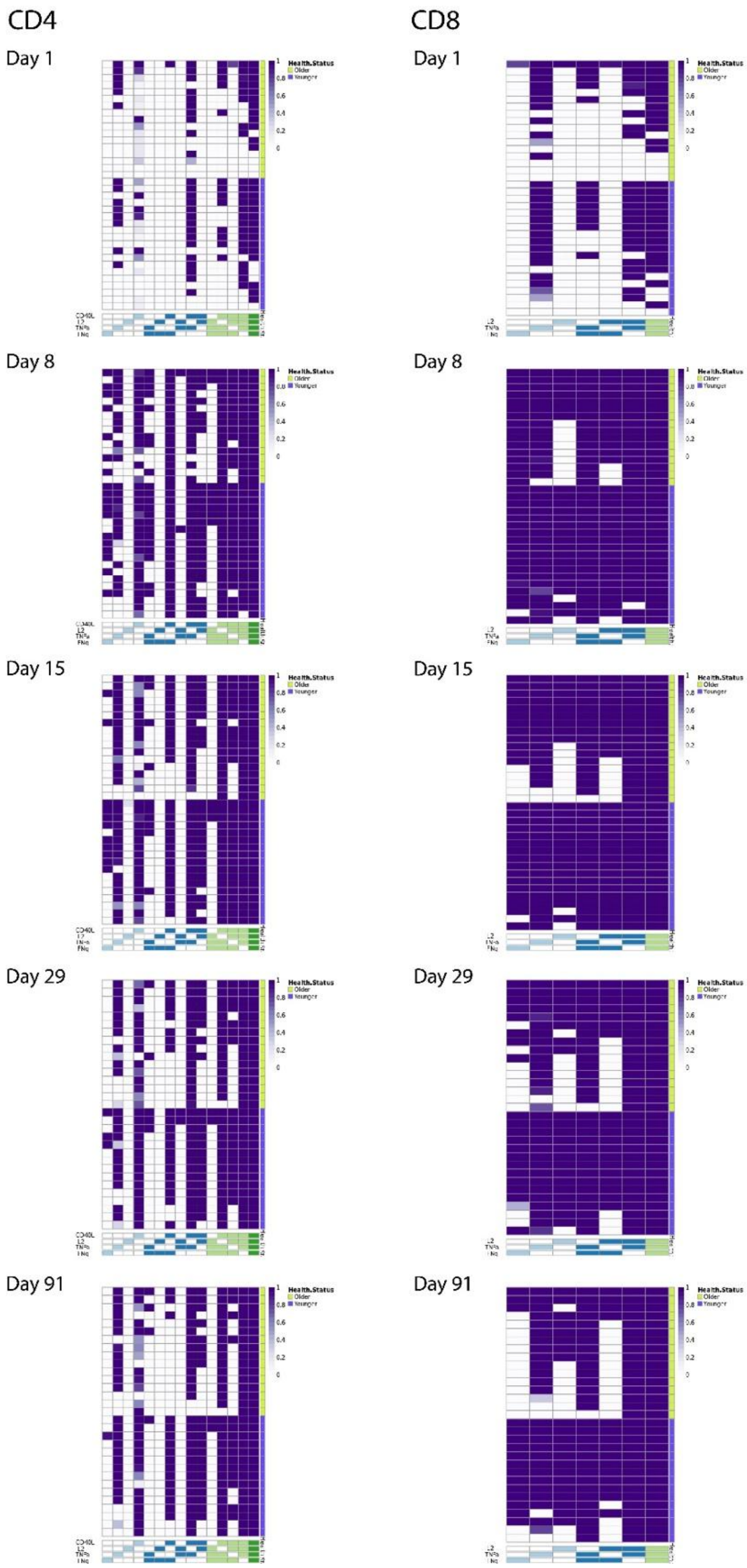

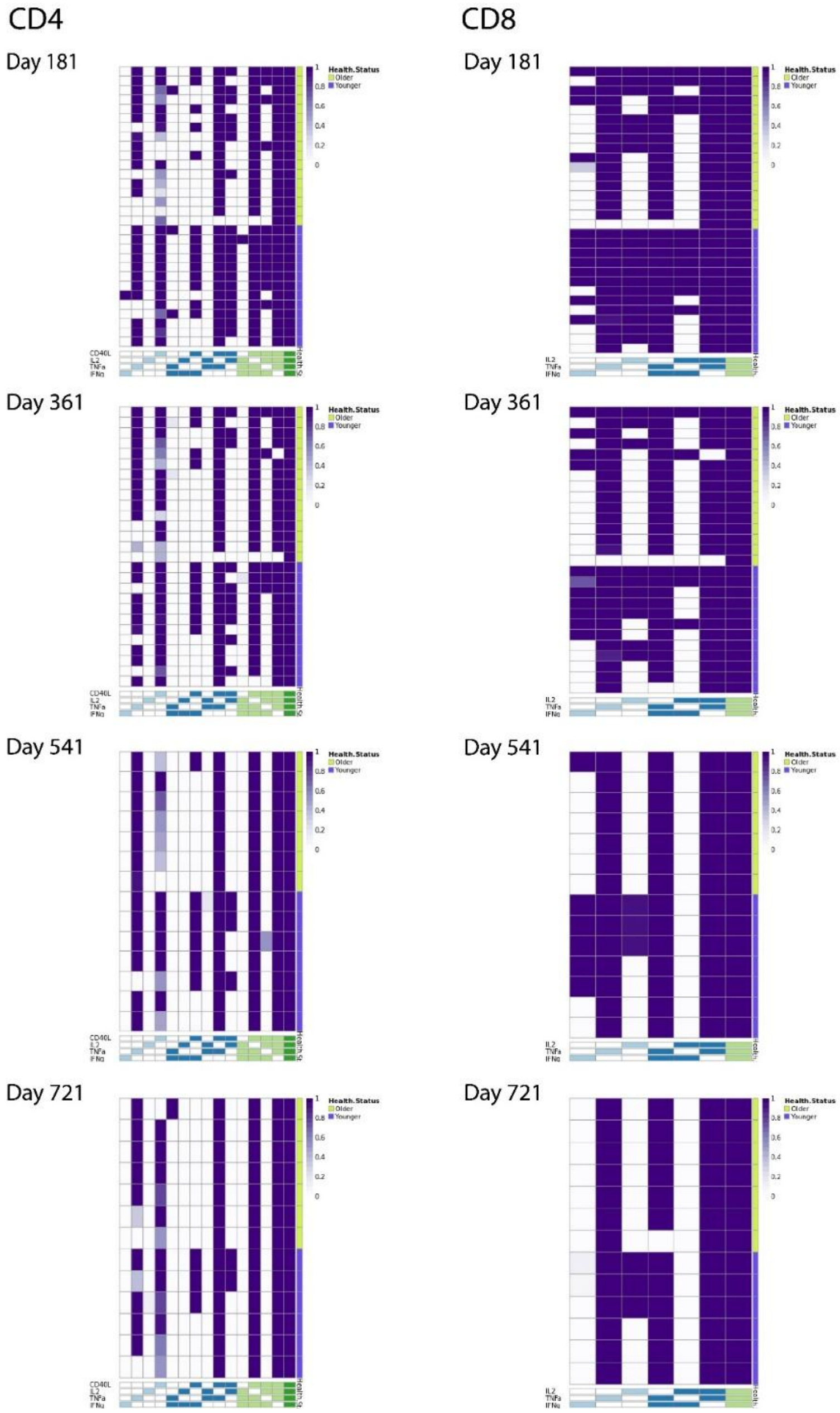
CD4
CD8
Day 181
Day 361
Day 541
Day 721
Health.Status
Older
Younger
CD40L
IL2
TNFa
IFNg
1
0.8
0.6
0.4
0.2
0

The heatmaps display posterior probabilities derived from COMPASS, based on ICS data comparing unstimulated (DMSO) and Pre-F peptide-stimulated conditions. In the heatmap, each column represents a non-overlapping T cell subset defined by a unique combination of cytokine expression, ordered from least to most polyfunctional (left to right: 1 to 5 functions; colored from light blue to green). Each row corresponds to a study participant, grouped by age (older adults at the top, younger adults at the bottom). Dark blue indicates a high probability that a given subset is antigen specific for that participant, while white indicates absence. T cell subsets with maximum posterior probabilities below 0.005 were excluded from the plots.

Fig. S4. Distribution of AIM+ T cells within UMAP and differential gene expression assay.

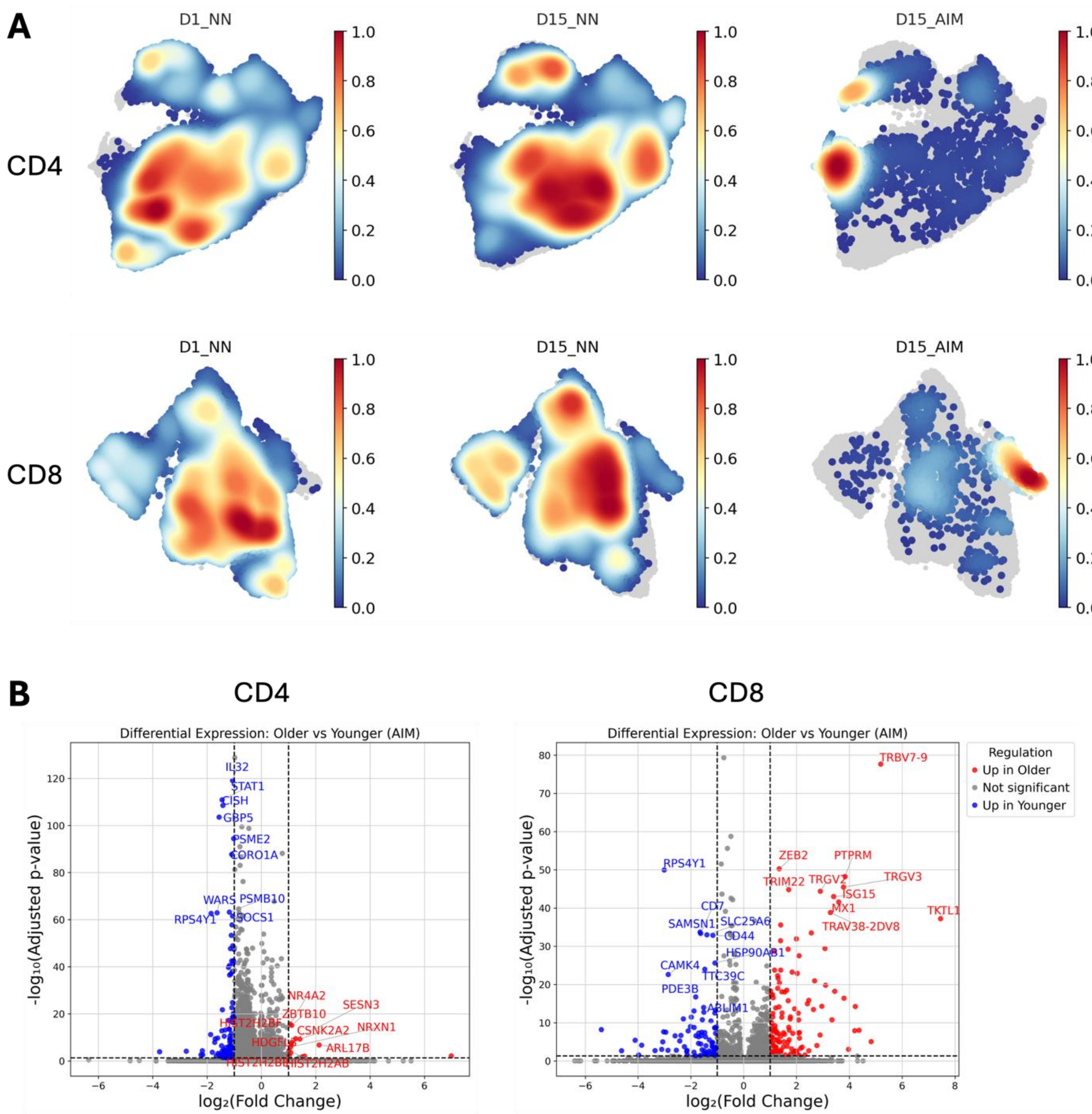

(**A**) Distributions of Day 1 (D1) non-naïve (NN), D15 NN, and D15 AIM+. Each panel displays the normalized cell density for a single group.

(**B**) Volcano plot of differentially expressed genes in Day 15 AIM^+ $CD8^+$ T cells.

Fig. S5. Clonal expansion by time and donor.

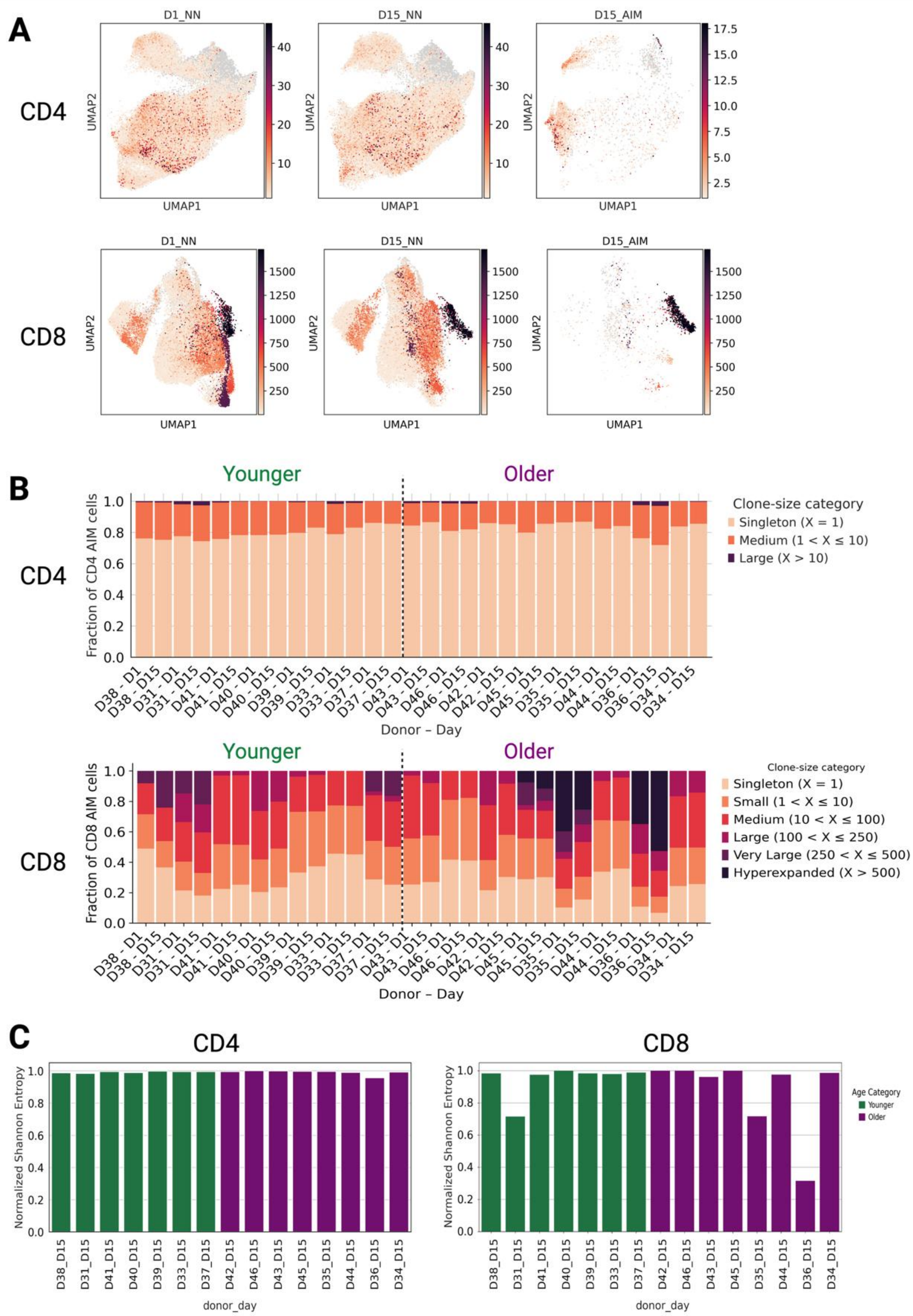

(**A**) UMAPs depicting clonal expansion of T cells based on amino acid sequence identity, stratified by Day 1 (D1) non-naïve (NN), D15 NN, and D15 AIM$^+$ conditions. Each point represents an individual cell, colored according to the size of its clonal family. The color scale reflects the number of cells in each clonotype (black = larger clone size; pale orange = smaller clone size). CD4$^+$ T cells are shown on the top panel, while CD8$^+$ T cells are shown on the bottom panel, with donors ordered from younger to older.

(**B**) Stacked bar plots illustrating the proportion of CD4$^+$ and CD8$^+$ T cells binned by clonal expansion category for D1 and D15 within each donor. Donors are ordered from younger to older individuals. Each bar represents an individual donor and day, with colors corresponding to the clonal expansion categories.

(**C**) Bar plot showing the Shannon diversity per donor for D15 AIM$^+$ CD8$^+$ T cells. Each bar represents one donor.

Table S1. Mutations (shown in bold) in the customized pre-F peptide pool compared to wild-type (WT).

**Amino Acids number in WT sequence**	**Sequence in WT vs customized Pre-F peptide pool**
Peptides 24-29	WT:<br>...LQLLMQSTPAT<br>**NNRARRELPRFMNYTLNNAKKTNVTLSKKRKRRFLGFLLGVGS**<br>AI**A**SGVAV**S**KVLHLEGEVNK...<br><br>Pre-F pool:<br>...LQLLMQSTPAT<br>**GSGS**<br>AI**C**SGVAV**C**KVLHLEGEVNK...
Peptides 35-38	WT:<br>...VSVLT**S**K...<br><br>Pre-F pool:<br>...VSVLT**F**K...
Peptides 40-42	WT:<br>...LLPI**V**NK...<br><br>Pre-F pool:<br>...LLPI**L**NK...
Peptides 59-61	WT:<br>...SYSIM**S**IIKE...<br><br>Pre-F pool:<br>...SYSIM**C**IIKE...
Peptides 79-82	WT:<br>...CDTMNS**L**TLPSE...<br><br>Pre-F pool:<br>...CDTMNS**R**TLPSE...
Peptides 100-103	WT:<br>...VGNTLY**Y**VNKQ...<br><br>Pre-F pool:<br>...VGNTLY**C**VNKQ...

Table S2. Antibodies used in ICS assay (https://rrid.site/).

Marker	Clone	Fluorochrome	Vendor	Catalog #	RRID
CD45RA	HI100	BUV395	BD	740298	AB_2740037
LIVE/DEAD	N/A	LIVE DEAD Blue	Thermo Fisher	L34962	N/A
CD69	FN50	BUV563	BD	748764	AB_2873167
TCR-gd	11f2	BUV661	BD	750019	AB_2874238
IL-5	TRFK5	BV421	Biolegend	504311	AB_2563161
IFN-g	B27	BV480	BD	566176	AB_2739573
IL-17A	BL168	BV605	Biolegend	512326	AB_2563887
TNF-α	Mab11	BV650	Biolegend	502938	AB_2562741
CD154	24-31	BV711	Biolegend	310838	AB_2563845
IL-4	MP4-25D2	BV786	BD	564113	AB_2738601
CD14	63D3	AF488	Biolegend	367130	AB_2721360
CD16	3G8	AF488	Biolegend	302019	AB_492974
CD19	HIB19	AF488	Biolegend	302219	AB_389313
CD56	5.1H11	AF488	Biolegend	362517	AB_2564092
CD4	SK3	cFluor B548	Cytek	R7-20043	N/A
CD8	SK1	PerCP	Biolegend	344708	AB_1967149
CD107a	H4A3	PE	Biolegend	328608	AB_1186040
CCR7	150503	PE-CF594	BD	562381	AB_11153301
CD3	SK7	PE-Fire 700	Biolegend	344864	AB_2876653
IL-13	JES10-5A2	PE-Cy7	Biolegend	501914	AB_2616746
IL-2	MQ1-17H12	AF647	Biolegend	500315	AB_493370
GZMB	QA16A02	AF700	Biolegend	372222	AB_2728389

Table S3. Antibodies used in AIM assay (https://rrid.site/).

Marker	Clone	Fluorochrome	Vendor	Catalog #	RRID
**CD14**	63D3	Alexa Fluor 488	Biolegend	367130	AB_2721360
**CD16**	3G8	Alexa Fluor 488	Biolegend	302019	AB_492974
**CD19**	HIB19	BUV563	BD Horizon	569676	AB_3685221
**CD56**	5.1H11	cFluor R720	Cytek	R7-20089	N/A
**CD3**	SK7	BUV496	BD	741206	AB_2870764
**CD4**	SK3	cFluor B548	Cytek	R7-20044	AB_3076337
**CD8**	SK1	BUV805	BD	612890	AB_2870178
**CD45RA**	HI100	BUV395	BD	740298	AB_2740037
**TCRγδ**	B1	PE-Fire 700	Biolegend	331237	AB_2876638
**CCR7/CD197**	150503	PE-CF594	BD	562381	AB_11153301
**CD154/CD40L**	24-31	Pacific Blue	Biolegend	310820	AB_830699
**CD107a**	H4A3	PE	Biolegend	328608	AB_1186040
**CD38**	HIT2	APC/Fire 810	Biolegend	303550	AB_2860784
**HLA-DR**	L243	BV711	Biolegend	307644	AB_2562913
**CXCR5 (CD185)**	RF8B2	Alexa Fluor 647	BD	558113	AB_2737606
**CD137/4-1BB**	4B4-1	BV480	BD	746700	AB_2743967
**OX40/CD134**	Ber-Act35	BV605	Biolegend	350028	AB_2629633
**CD25/IL-2R**	BC96	BV785	Biolegend	302638	AB_2563808
**PD-L1**	29E.2A3	PE/Fire 810	Biolegend	329755	AB_2894668
**CD95**	DX2	BB700	BD	566542	AB_2869780
**CXCR3 (CD183)**	G025H7	BV421	Biolegend	353716	AB_2561448
**CCR6 (CD196)**	11A9	PE-Cy7	BD	560620	AB_1727440
**PD-1 (CD279)**	EH12.2H7	BV650	BD	564104	AB_2738595
**CCR4 (CD194)**	1G1	BUV661	BD	752522	AB_2917512
**ICOS**	DX29	BUV737	BD	749665	AB_2873929
**LIVE/DEAD Blue**	N/A	UV BLUE	Thermo	L34962	N/A
**CD69**	FN50	APC/Fire 750	Biolegend	310946	AB_2616709
